# Comparison between hCG and GnRH Agonist for Ovulation Trigger in GnRH Antagonist *In-Vitro* Fertilization Cycles in a Tertiary Hospital in Malaysia: An observational study

**DOI:** 10.5935/1518-0557.20230066

**Published:** 2024

**Authors:** Nor Azimah Aziz, Adibah Ibrahim, Roziana Ramli, Nasuha Yaacob, Siti Nabillah Abdul Rahman, Engku Husna Engku Ismail, Ahmad Akram Omar

**Affiliations:** 1 Department of Obstetrics and Gynaecology, School of Medical Sciences, Universiti Sains Malaysia, 16150 Kubang Kerian, Kelantan, Malaysia; 2 Department of Obstetrics and Gyneaecology, Hospital Universiti Sains Malaysia, 16150 Kubang Kerian, Kelantan, Malaysia; 3 Department of Obstetrics and Gynaecology, Hospital Sultanah Nur Zahirah, Kuala Terengganu, Terengganu, Malaysia

**Keywords:** ovulation trigger, hCG-trigger, GnRH agonist-trigger, OHSS

## Abstract

**Objective:**

hCG is commonly used as an ovulation trigger in IVF. Its usage is associated with OHSS. GnRH agonist is an alternative to hCG and is associated with reduced incidence of OHSS. This study compared the cycle outcomes of GnRH agonists with hCG as an ovulation trigger in IVF cycles.

**Methods:**

The medical notes of 209 IVF cycles receiving GnRH agonist and hCG as ovulation trigger over 18 months were reviewed in this retrospective study. The number and quality of mature oocytes, the number and quality of embryos, pregnancy rates, and outcomes were compared using Independent T-test or One-way ANOVA for normal distribution. The Mann-Whitney test or Kruskal-Wallis test was used for not normally distributed. *p*<0.05 was considered statistically significant.

**Results:**

The cycle outcomes of 107 GnRH agonist-trigger and 102 hCG-trigger were compared. The MII oocytes retrieved and 2PN count was significantly higher in the GnRH agonist trigger group (*p*<0.001). Clinical pregnancy rate and ongoing pregnancy were higher in the GnRH agonist trigger group but were not statistically significant. The GnRH agonist trigger group was associated with low OHSS than the hCG trigger group (n=2(1.9%) and n=12(11.8%) respectively, *p*=0.004).

**Conclusion:**

GnRH agonist trigger is an option as a final maturation trigger in high-responder women undergoing IVF or ICSI cycles.

## INTRODUCTION

The use of any assisted reproductive technique, particularly In Vitro Fertilization (IVF) and intracytoplasmic sperm injection (ICSI), is becoming more frequent. Unlike ovarian stimulation in intrauterine insemination (IUI), which targeting for no more than two follicular growth, ovarian stimulation in these procedures requires adequate follicular development and maturation, best achieved with unfractionated Follicle Stimulating Hormones (FSH) with or without additional Luteizing Hormones (LH) (van Rumste *et al*., 2008; Delvigne & Rozenberg, 2002). Undoubtedly, the incidence of ovarian hyperstimulation syndrome (OHSS) is closely related to the type of ovarian stimulating agent, highest in unfractionated FSH stimulating protocol (Yang *et al*., 2021).

OHSS could be a potentially life-threatening condition that causes doctors significant challenges (Humaidan *et al*., 2010). The increasing ovarian size during the initial form of OHSS causes abdominal discomfort and progresses to abdominal distension, pain, nausea, vomiting, and diarrhea. As the condition advances, there will be extravascular protein-rich exudates in the abdominal cavity, peritoneum, pleural, and even in the pericardiac space, causing intravascular volume depletion and depletion and hemoconcentration, oliguria, electrolyte imbalance, and end-organ failure. The thromboembolic phenomenon is the ultimate complication of OHSS which may be fatal (Humaidan *et al*., 2010; Cluroe & Synek, 1995; Mozes *et al*., 1965). Mild OHSS accounts for 20% to 33% of the IVF cycle following ovarian hyperstimulation (Humaidan *et al*., 2013; Yen *et al*., 1968). The more worrying condition is severe and critical OHSS, a life-threatening condition, which accounts for 10-20% of the high-risk population (Kupka *et al*., 2014; Papanikolaou *et al*., 2006).

Human chorionic gonadotrophin (hCG) is the main culprit for OHSS. During the final stage of oocyte maturation, the LH surge triggers the rupture of matured follicles to expel the oocytes into the fallopian tubes. hCG, which has considerable structural similarities with LH, is commonly used to simulate LH surge in the IVF/ICSI cycle. The granulosa cells of the hyperstimulated ovaries produce vasoactive cytokines such as vascular endothelial growth factor (VEGF) and express mRNA for VEGF. VEGF increases vascular permeability (El Tokhy *et al*., 2016; Imoedemhe *et al*., 1991). Exogenous hCG administration increases VEGF expression in granulose-lutein cells (Wang *et al*., 2002; Chen *et al*., 2011; Le Gouez *et al*., 2011). The increased number of oocytes and upregulation of VEGF in luteinized granulosa cells by hCG increases the serum VEGF level, resulting in OHSS manifestations.

Triggering final oocyte maturation with GnRH agonist in ovarian stimulation is beneficial when premature LH is inhibited in the GnRH antagonist IVF/ICSI cycle as an alternative to hCG to reduce the risk of OHSS. Several studies reported that more mature oocytes were retrieved after the GnRH agonist trigger, an effect of a more physiological surge of FSH and LH (Oktay *et al*., 2010; Imoedemhe *et al*., 1991). However, reports on the outcomes of IVF/ICSI using GnRH agonists as ovulation triggers, especially among PCOS patients, are conflicting. There were concerns about lower pregnancy rates with the use of GnRH agonists as ovulation triggers (Alyasin *et al*., 2016; Humaidan *et al*., 2005; Kolibianakis *et al*., 2005; Youssef *et al*., 2011) as GnRH agonist cause suboptimal yields of mature oocytes (Honnma *et al*., 2011; Castillo *et al*., 2012), leading to the development incapability of embryos (Beckers *et al*., 2003; Humaidan & Polyzos, 2014).

The present study aimed to evaluate the effect of GnRH agonists on the outcomes of IVF/ICSI triggered by GnRH agonists. Its effect on oocyte maturity, quality of the embryo, pregnancy rates, and OHSS developed was compared with the standard hCG-triggered protocol.

## MATERIALS AND METHOD

This retrospective observational study involved all women who underwent IVF/ICSI cycles using GnRH antagonist protocol from January 2016 until December 2020. The study protocol was approved by the National Medical Research Committee (NMRR-21-1024-59576) and the Human Research Ethics Committee USM (JEPeM Code: USM/JEPem/21040343). The data from 209 normogonadotrophic patients were reviewed. Patients aged more than 25 and less than 40 years, with BMI between 18kg/m^2^ and less than 30kg/m^2^, having FSH and LH baseline levels less than 10miu/ml were included in the study. Patients with hyperprolactinemia, diabetes mellitus, and medical disorders that contraindicated pregnancy were excluded. The sociodemographic data, the treatment outcome between hCG and GnRH agonist triggers in GnRH antagonist IVF/ ICSI cycles, and other related details were retrieved from the case notes.

### Participants

The study cohort consisted of all patients who underwent IVF/ ICSI cycles receiving either hCG or GnRH agonists as ovulation triggers.

### The protocol

All patients received daily gonadotrophin injections starting from the third day of menses. The starting dose of gonadotrophins ranged from 150iu to 300iu, depending on the patient’s requirement. The growth of the follicles was monitored using a serial transvaginal ultrasound. When two or more (≥2) leading follicles were at least 12mm in mean diameter, the GnRH antagonist (Orgalutran^®^, 0.25mg daily, MSD; or Cetrotide^®^ 0.25mg daily, Merck) was given. The final oocyte maturation was triggered when three or more (≥3) follicles were at least 18mm in mean diameter, using either subcutaneous 10,000iu hCG (Pregnyl^®^, MSD; or Hucog^®^, Firstline) or subcutaneous 0.25mg Ovidrel^®^, Serono; or GnRH agonist (Decapeptyl^®^ 0.2mg, Ferring).

Transvaginal ultrasound-guided oocyte retrieval was performed 35 to 35.5 hours after ovulation trigger injection using a single lumen retrieval needle.

Fertilization was checked 18h after insemination by the appearance of two pronuclei. The embryos were graded according to their morphology using the Istanbul consensus: Grade 1 (Good): <10% fragmentation, stage-specific cell size, and no multinucleation. Grade 2 (Fair): 10-25% fragmentation, stage-specific cell size for most cells, and no evidence of multinucleation. Grade 3 (Poor): severe fragmentation (>25%), cell size not stage-specific, and evidence of multinucleation. Grade 1 and 2 embryos were considered good-quality embryos, while Grade 3 embryos were poor-quality embryos.

Embryo transfer (ET) was performed under ultrasound guidance using a Cooks catheter (K-JETS-7017-SIVF, Cook Medical, Syndey IVF). The luteal phase was supported with vaginal progesterone and estradiol until 12 weeks of gestation if the pregnancy was achieved.

### Outcome measures

Serum beta hCG of more than 2miu/L taken 14 days post-ET confirmed implantation, termed as biochemical pregnancy. An ultrasound was performed at seven weeks post-ET. Visualization of gestational sac confirmed clinical pregnancy. The occurrence of OHSS and its severity should it happen, were documented. OHSS was classified as mild, moderate, severe, and critical according to its symptoms and biochemical findings (Humaidan *et al*., 2016).

### Statistical analysis

The researchers analyzed the data using SPSS version 27.0. In the descriptive analysis, all numerical data were expressed as mean and standard deviation (SD), while categorical data were expressed as frequency (n) and percentage (%).

The oocyte maturity rate was calculated by dividing the MII oocytes by the total oocytes retrieved, multiplied by 100% (ESHRE Special Interest Group of Embryology & Alpha Scientists in Reproductive Medicine, 2017).

The fertilization rate was expressed as the number of inseminated/injected oocytes fertilized (presence of 2PN after injection) to the number of the oocytes inseminated multiple with 100% (ESHRE Special Interest Group of Embryology & Alpha Scientists in Reproductive Medicine, 2017).

The clinical pregnancy rate was calculated as the number of cases with evidence of at least one gestational sac by TVS, divided by the number of transfers.

Statistical differences were evaluated using the Independent T-tests or Fischer Exact test whenever indicated.

*p*<0.05 was considered statistically significant.

## RESULTS

### Patient characteristics

Of 209 women receiving IVF/ICSI treatment, 107 women (51.2%) received GnRH agonist trigger, and 102 women (48.8%) received hCG trigger. There was no difference in the age, duration, and cause of subfertility and baseline FSH, LH, and progesterone levels on trigger day. The mean antral follicle counts (AFC) in both groups were more than 10, being 15.37 (SD 6.85) in the GnRH agonist trigger group and 11.82 (SD 4.52) in the hCG trigger group (*p*<0.001). As expected, there was also a significantly higher estradiol (E2) level in the GnRH agonist group (19822.58 (SD 10467.22); *p*<0.001) than in the hCG trigger group (Table 1).

**Table 1:** Comparison of the personal and cycle characteristics between the GnRH agonist trigger group and hCG trigger group.

Variables	Mean (SD)		*p*-value
	hCG (n=102)	GnRH Agonist (n=107)	
Age (years)^a^	32.40 (4.15)	32.31 (4.37)	0.874^c^
BMI (kg/m^2^)^a^	23.90 (3.38)	24.06 (3.28)	0.724^c^
Duration of infertility^a^	6.48 (3.55)	6.25 (3.37)	0.634^c^
Duration of stimulation (days)^a^	10.9 (1.13)	9.82 (0.86)	0.055^c^
Total dose of gonadotropins (IU)^a^	2160.66 (646.16)	2066.36 (561.03)	0.261^c^
Baseline FSH level^a^	6.96 (2.28)	6.65 (1.89)	0.298^c^
Baseline LH level^a^	5.29 (2.61)	5.64 (3.56)	0.425^c^
E2 level on trigger day^a^	13081.68 (9634.67)	19822.58 (10467.22)	<0.001^* c^
P4 level on trigger day^a^	3.49 (1.84)	4.00 (4.05)	0.264^c^
AFC^a^	11.82 (4.52)	15.37 (6.85)	<0.001^* c^

HCG=human chorionic gonadotropin; GnRH=gonadotropin receptor hormone; BMI=body mass index; FSH=follicle stimulating hormone; LH=luteinizing hormone; AFC=atrial follicle count

* *p*<0.05;

a Data presented in mean (SD)

b Data presented in frequency and percentage; n (%)

c Independent T-test

d Pearson’s Chi-Square Test

### Stimulation outcome

The mean number of follicles triggered on the trigger day was 20.16 (SD 9.29) in the GnRH agonist group, which was significantly higher than the hCG trigger group (*p*<0.001). The number of retrieved oocytes was also significantly more (13.36 - SD 7.47) in the GnRH agonist group and 8.88 (SD 5.93) in the hCG group, *p*<0.001) (Table 2). The GnRH agonist group also had more oocytes with 2PN (9.19 - SD5.40) as compared to 6.82 (8.33) in the hCG trigger group). This difference was statically significant with *p*=0.015.

**Table 2:** Comparison of cycle outcome between GnRH agonist trigger and hCG trigger.

Variables	Mean (SD)		*p*-value
	hCG (n=102)	GnRH Agonist (n=107)	
Total follicles on trigger day ^a^	12.05 (6.18)	20.16 (9.29)	<0.001 ^*c^
Number of oocytes retrieved ^a^	10.27 (6.10)	15.94 (7.96)	<0.001 ^*c^
Number of MII oocytes ^a^	8.88 (5.93)	13.36 (7.47)	<0.001 ^*c^
Oocytes maturity rate (%) ^a^	86.70 (15.54)	87.50 (13.00)	0.685 ^c^
Number of oocytes inseminated ^a^	8.88 (5.93)	13.36 (7.47)	<0.001 ^*c^
2PN ^a^	6.82 (8.33)	9.19 (5.40)	0.015 ^*c^
Fertilization rates (%) ^a^	71.71 (18.99)	70.14 (19.01)	0.551 ^c^
Total Embryo ^a^	6.03 (4.05)	9.01 (5.40)	<0.001 ^*c^
Top quality embryo ^a^	2.89 (3.15)	2.98 (3.44)	0.845 ^c^
Embryo utilized ^a^	5.14 (3.46)	6.62 (4.35)	0.007 ^*c^
Embryo utilization rates (%) ^a^	87.74 (16.50)	75.13 (21.29)	<0.001 ^*c^
Clinical pregnancy rate ^b^	39 (38.2)	44 (41.1)	0.670 ^d^
Ongoing pregnancy rate ^b^	32 (31.4)	41 (38.3)	0.292 ^d^
Miscarriage rates (%) ^a^	5.23 (17.70)	2.65 (14.69)	0.254 ^c^

HCG=human chorionic gonadotropin; GnRH=gonadotropin receptor hormone; BMI=body mass index; FSH=follicle stimulating hormone; LH=luteinizing hormone; AFC=atrial follicle count

* *p*<0.05;

a Data presented in mean (SD)

b Data presented in frequency and percentage; n (%)

c Independent T-test

d Pearson’s Chi-Square Test

### Clinical outcome

On a per-cycle basis, the fertilization rate, clinical pregnancy rate, ongoing pregnancy, and miscarriage rate were similar between the two groups. The fertilization rate in the GnRH agonist trigger group was 70.14% (SD 19.01%), while it was 71.71% (SD 18.99%) in the hCG trigger group (*p*>0.05). Although there was a higher clinical pregnancy rate in the GnRH agonist trigger group (44.0% - SD 41.1%) and 39% (SD 38.2%) for the hCG trigger group), the difference was not statistically significant (*p*>0.05) (Table 2). As all the women in this study were high responders, they were at risk for OHSS. We compared the incidence of OHSS between the two studied groups. Triggering the ovulation with GnRH agonist significantly reduces OHSS (*p*<0.001). Only two women (1.9%) in the GnRH agonist group, while twelve women (11.8%) in the hCG trigger group experienced it (Table 3). All women had either mild or moderate OHSS. None of them experienced severe or critical OHSS.

**Table 3:** Comparison of the association of OHSS between the GnRH agonist trigger and hCG trigger group.

Variable	OHSS n (%)		X^2^ (df)	*p*-value^¥^
	No	Yes		
**Trigger Group** hCG GnRH Agonist	90 (88.2) 105 (98.1)	12 (11.8) 2 (1.9)	8.182 (1)	0.004^*^

¥ Pearson’s Chi-square test n=frequency; %=percentage

OHSS=ovarian hyperstimulation syndrome; HCG=human chorionic gonadotropin; GnRH=gonadotropin receptor hormone

* *p*<0.05

## DISCUSSION

The present study was performed among high-responder women, as shown by the high AFC and estradiol levels in both groups. The increase in the estradiol level resulted from excessive response to ovarian hyperstimulation, which increased the release of VEGF. VEGF synthesis is an essential part of this pathological condition. hCG stimulates VEGF from granulosa cells via its long-lasting, sustained LH-like activity (6-10 days). hCG trigger is a prerequisite for OHSS development. Substituting it with a GnRH agonist will eliminate the risk of OHSS due to its shorter half-life LH surge induced by GnRH agonist (24h) (Casper, 2015). Over the past decade, GnRH agonists used during the final oocyte maturation effectively reduced OHSS. Only two women in the GnRH agonist trigger group developed OHSS in this study, in contrast to twelve women who did so in the hCG trigger group. The findings of this study echoed many other studies (Yilmaz *et al*., 2020; Iliodromiti *et al*., 2013; Radesic & Tremellen, 2011; DiLuigi *et al*., 2010). Datta *et al*. (2014) found a 16.2% OHSS with GnRH agonist trigger and 31.0% with hCG trigger. There was one case of severe OHSS in the hCG trigger group. Like in this study, no severe OHSS found in the GnRH agonist group.

A Cochrane review in 2011 and 2014 recommended not to use GnRH agonist as a routine trigger agent in a fresh autologous cycle, as it lowered the live birth and ongoing pregnancy rates (Youssef *et al*., 2011; 2014). The negative impacts were caused by premature luteolysis of the corpus luteum and luteal phase insufficiency among the patients. The present study found that the number of retrieved and MII oocytes was significantly higher in the GnRH agonist group than in the hCG trigger group. The consistent surge in both FSH and LH caused by GnRH agonists mimics the natural cycle, thus inducing oocyte maturation and yielding more mature oocytes (Krishna *et al*., 2016; Oktay *et al*., 2010; Erb *et al*., 2010; Humaidan *et al*., 2005). Many authors have concluded that LH should work in accordance with FSH for optimal oocyte maturation. Subsequent clinical trials reported a higher proportion of mature oocytes after the GnRH agonist trigger than hCG, similar to the present study’s findings (Humaidan *et al*., 2011; Lin *et al*., 2013; Reddy *et al*., 2014). On the contrary, some others have found nonsignificant results (Engmann *et al*., 2008; Melo *et al*., 2009; Bodri *et al*., 2011). The presence of more 2PN oocytes in this study was consistent with the maturity of those oocytes, causing them to be more capable of fertilizing.

The present study found a higher pregnancy rate per cycle in the GnRH agonist trigger group than the hCG trigger group, although the difference was not statistically different. The concern of premature luteinization with the short half-life of GnRH agonist during final oocyte maturation may cause lower successful outcomes in the GnRH agonist trigger group. The traditional practice of hCG administration as luteal phase support will induce OHSS, especially among the high-responders. However, Datta *et al*. (2014) in their study on sixty-two women who underwent GnRH agonist trigger with low dose hCG for luteal phase support, reported none developed severe OHSS. All ten women who had OHSS were in the mild OHSS group and received only outpatient treatment.

GnRH agonist was shown to be effective as luteal phase support (Haahr *et al*., 2017; Pirard *et al*., 2006), evidenced by higher levels of LH, clinical pregnancy rate, and low miscarriage rates. Besides GnRH agonists, progesterone supplementation via intramuscular injections or vaginal pessaries gave similar benefits.

## CONCLUSION

The present study concluded that GnRH agonist is effective and safe as a final oocyte maturation agent among high responders without significantly affecting the clinical and ongoing pregnancy rates. Our data reassures us that GnRH agonists can reduce OHSS, in addition to other methods of OHSS prevention. We recommend more extensive prospective studies to investigate how to increase the clinical pregnancy rates using GnRH agonists as luteal phase support.
